# Effects of Blueberry Consumption on Preference, Digestibility, and Oxidative Balance in Dogs

**DOI:** 10.3390/ani15101502

**Published:** 2025-05-21

**Authors:** Marta Maturana, Lorena Castillejos, Eduard Jose-Cunilleras, Miquel Montserrat-Malagarriga, Juan Alcaraz, Jose García, Susana M. Martín-Orúe

**Affiliations:** 1 Animal Nutrition and Welfare Service (SNiBA), Department of Animal and Food Science, Universitat Autònoma de Barcelona (UAB), 08193 Bellaterra, Spain; marta.maturana@uab.cat (M.M.); susana.martin@uab.cat (S.M.M.-O.); 2 Department of Animal Medicine and Surgery, Universitat Autònoma de Barcelona (UAB), 08193 Bellaterra, Spain; eduard.jose.cunilleras@uab.cat; 3 Visán Industrias Zootécnicas SL, 28500 Madrid, Spain; juan.alcaraz@visan.es (J.A.); tecnico@visan.es (J.G.)

**Keywords:** dogs, antioxidants, blueberries, preference, digestibility, oxidative stress, exercise, Beagles

## Abstract

The growing awareness of the diet–health connection drives interest in natural dog diets, which replace synthetic additives like antioxidants with natural ingredients. In Trial 1 of this study, preference for diets containing powdered *Fucus* algae (1.5%), powdered clove (0.45%), or whole blueberries (3%) was evaluated using the two-bowl method. Dogs exhibited a clear preference for the blueberry diet. In Trial 2, the impact of blueberries on apparent digestibility was assessed. Twelve Beagles were fed either a control diet (CON) or the same diet with 3% blueberries (BLU). No differences were observed, except for cellulose digestibility. Trial 3 evaluated the effect of blueberries on oxidative status during submaximal exercise. The same dogs were fed either CON or BLU diets for 4 weeks, with eight dogs completing treadmill exercises at the start and end of this period. Blood samples were collected before and after exercise. Trial 3 was replicated in summer and winter, reversing diet groups. Exercise increased creatine kinase (CK), urea, and malondialdehyde and decreased glutathione peroxidase, with stronger effects in summer, suggesting heat stress. Although the BLU diet did not mitigate exercise-induced changes, lower resting CK levels after 4 weeks in summer suggest protection against heat stress, warranting further study.

## Simple Summary

Dog owners are increasingly concerned about the impact of diets on their pets’ health, driving demand for natural alternatives. This study investigated the effects of adding blueberries to a wet commercial diet on oxidative balance in dogs subjected to a controlled exercise. Blueberries were selected over other natural antioxidant ingredients, such as *Fucus* algae or clove, due to their superior palatability and minimal impact on digestibility. To evaluate its effects on oxidative status, dogs were fed either a control diet or the same diet containing blueberries for 4 weeks. Animals performed two treadmill exercises to induce oxidative stress, at the start and end of the experimental period. Blood biomarkers of muscle activity, inflammation, and oxidative status were analyzed. The trial was conducted in both summer and winter. Exercise significantly influenced markers of muscle activity, oxidative stress, and antioxidant enzymes, with more pronounced responses in summer, suggesting a relevant impact of the environmental temperature. No significant differences in biomarker responses to exercise were observed between diets. However, lower creatine kinase levels were found at rest in the blueberry-fed group during summer. These results may indicate a protective effect of blueberries against heat stress, warranting further investigation.

## 1. Introduction

Reactive oxygen species (ROS) are typically by-products of physiological processes linked to aerobic metabolism, with the major endogenous sources being the mitochondrial electron transport chain and NADPH oxidases [1]. In addition to physiological processes, other sources of ROS include exposure to air pollutants, industrial chemicals, UV radiation, and exercise [2,3,4,5]. The highly reactive nature of these molecules makes them potentially damaging to proteins, lipids, and nucleic acids. However, endogenous mechanisms regulate ROS levels to maintain a dynamic equilibrium, involving enzymes such as glutathione peroxidases (GPX), as well as non-enzymatic pathways [6,7].

An imbalance between ROS production and removal toward their formation can still occur, inducing what is known as oxidative stress. In dogs, oxidative stress has been linked to processes such as carcinogenesis [8], brain aging [9], heart disease [10], and anemia [11]. Research on strategies to counteract oxidative stress in dogs primarily focuses on blocking ROS generation, scavenging existing ROS, and enhancing endogenous antioxidants [12]. Dietary antioxidants can provide daily protection against oxidative stress. With the increasing trend among pet owners of choosing natural diets, the search for natural antioxidants becomes important, for both health benefits and food safety [13]. The range of ingredients with antioxidant potential is vast [14]; however, the scientific evidence behind some of these ingredients is still developing.

One group of compounds with known antioxidant properties is polyphenols, which can be found in plant-based foods such as fruits and vegetables. The mechanisms associated with their antioxidant capacity include hydrogen atom donation, inhibition of enzymes involved in ROS production, or metal ion chelation [15]. Berries are a rich source of these molecules, with in vitro and in vivo studies pointing at their antioxidant, anti-inflammatory, antihypertensive, microbiota modulation, and cell-protecting effects [16]. Berry polyphenols are classified based on their structural characteristics into flavonoids, phenolic acids, stilbenes, tannins, and lignans [17]. Among flavonoids, anthocyanins are responsible for the vibrant colors of these fruits but also the main contributors to the total antioxidant capacity of blueberries and black currants [18]. Spices and herbs are another group of natural ingredients that possess antioxidant activity due to their polyphenol content. In this case, the main phenolic groups responsible for their antioxidant properties are phenolic acids, phenolic diterpenes, flavonoids, and volatile oils, such as eugenol [19]. Cloves, for example, have traditionally been used as food preservatives, antioxidants, and antimicrobials, representing one of the richest sources of eugenol and gallic acid [20]. Among the newest natural ingredients being explored for use in pet food are aquatic plants, such as macroalgae and microalgae, which represent a rich source of antioxidants. One of the most prominent brown macroalgae is *Fucus vesiculosus*, whose content in phenolic compounds such as phlorotannins and flavonoids seems to be higher than those of other seaweeds [21].

To evaluate the potential benefits of diets or feed additives in ameliorating oxidative stress in dogs, challenges promoting an oxidant state can be employed. Exercise increases oxygen uptake and can induce oxidative stress, mainly due to increased oxidant production by the contraction of the skeletal muscle tissue [22]. The impact of exercise on dogs has been previously studied, mostly focusing on athletic dogs such as sled, hunting, or working dogs, which have different fitness and metabolic needs compared with other types of dogs, such as Beagles. Models of exercise-induced oxidative stress might hold value as an evaluation tool for antioxidants. Indeed, although still scarce, there are published studies in dogs where this kind of models has been used to assess the protective effects of α-tocopherol, β-carotene, and lutein, mainly in sport dogs such as sled dogs and Foxhounds [23,24,25,26], and only one example in Beagles [27].

The hypothesis of the present study was that the inclusion of selected natural ingredients with antioxidant potential in a diet for dogs could reduce the levels of markers of oxidative stress. Beyond the potential antioxidant benefits that these natural ingredients may offer to dogs, it is essential to assess their effects on digestibility and, ultimately, on preference, as these factors can determine the success of a new diet in the market. Therefore, this study began with the evaluation of the effect on preference of the inclusion of powdered clove, powdered *Fucus* algae, or whole blueberries in a wet diet for dogs. Since animals exhibited a clear preference for the blueberry diet, the second phase focused on assessing the potential impact of blueberries on apparent digestibility and, finally, on the apparent oxidative status of the dogs. For the oxidative study, a model of exercise-induced oxidative stress was employed, and the responses of various biomarkers related to muscle activity, inflammation, and oxidative balance were analyzed. Additionally, this research aimed to expand the understanding of exercise models in untrained Beagles as a tool for evaluating food additives, with a particular focus on antioxidants.

## 2. Materials and Methods

The use of animals, procedures, and sampling of this study were approved by the Animal Experimentation Ethics Committee of the Universitat Autònoma de Barcelona (UAB), following the European Union guidelines for the ethical care and handling of animals under experimental conditions (protocol number CEEAH 3281; DACAAR 11555).

### 2.1. Animals and Housing

A total of 12 adult Beagle dogs (6 females and 6 males) were included in this study, with an average body weight of 13.8 ± 1.96 kg and age of 3.1 ± 0.62 years. The animals were housed at the Experimental Farms Unit of the UAB, where 12 individual metal bar paneled kennels, each of them with a platform and an ad libitum fresh water point, were connected by a common corridor with access to an outdoor area. The dogs were housed in a single group, only individualized during feeding, preference tests, and total fecal collection days. Except for preference test days (see below), dogs were fed once daily in the morning (09:00–10:00 h), with rations calculated according to their individual maintenance energy requirements (MER), based on historical feed intake records and the metabolizable energy values declared by the food manufacturer. Dogs were weighed every 2 weeks, and adjustments to the daily ration were made if needed to maintain body weight. All animals underwent clinical and physical examinations and were deemed healthy prior to the start of the study.

### 2.2. Trial 1: Preference

#### 2.2.1. Diets and Experimental Design

The diets used in the preference trial were a wet control diet (CON) (20% dry matter), mainly composed of fresh chicken (65%), minerals, vitamins, linseed, glucosamine, chondroitin, inulin, and hydrolyzed yeast cell wall, and variations of this same diet including clove powder (0.45%) (CLO), *Fucus* algae powder (1.5%) (ALG), or whole blueberries (3%) (BLU). The inclusion levels were selected based on the range in which these ingredients are used in commercial feeds, as well as on previous publications, generally choosing concentrations within the upper end of this range. All diets were formulated by the manufacturer (Visán Industrias Zootécnicas SL, Madrid, Spain), trying to be similar in energy, crude protein, crude fat, and crude fiber content, with calculated values of 97 kcal/100 g, 11.50–11.60%, 4.25%, and 0.75%, respectively.

Palatability was assessed using a two-bowl test methodology, after validation of the animals’ ability to discriminate between foods [28]. Diets were compared in pairs, with a total of 6 comparisons: CON vs. CLO, CON vs. ALG, CON vs. BLU, CLO vs. ALG, CLO vs. BLU, and ALG vs. BLU. For each comparison, two recipients, each containing the amount of food required to meet the dogs’ daily ME requirements, were offered to the dogs for 7.5 min in the morning. Each comparison was conducted over 2 consecutive days, with the position of the bowls switched on the second day to avoid any positional bias. The 12 dogs underwent the 6 comparisons, which resulted in a total of 12 days of preference tests. The order in which the dogs performed the 6 comparisons was randomized using Excel’s function rand(), only ensuring that each combination was being tested every day by 2 dogs. The amount of food offered and the food leftovers were registered to calculate the food intake from each bowl by difference. Additionally, the first bowl the animal approached and the bowl from which the animal first consumed for more than 15 consecutive seconds were registered as first approach and first choice, respectively.

#### 2.2.2. Calculations and Statistical Analysis

The intake ratio (IR) (%) for the diets in each comparison (diet A vs. diet B), for each dog, was calculated as

$$IR_{\mathrm{diet}\;A}=\frac{\mathrm{intake}\;\left(g\right)\;\mathrm{of}\;\mathrm{diet}\;A}{\mathrm{intake}\;\left(g\right)\;\mathrm{of}\;\mathrm{diet}\;A+B}\;\cdot \;100$$

Intake ratios were analyzed using RStudio software (version 1.4.1106), with a Student’s *t*-test analysis following normality assessment via the Shapiro–Wilk test. First approach and first choice were analyzed using a Chi-square test. Significance was set at *p* < 0.05, and trends were considered at 0.05 ≤ *p* ≤ 0.10.

### 2.3. Trial 2: Digestibility

#### 2.3.1. Diets and Experimental Design

The most preferred BLU diet (Trial 1) and the CON diet were assessed in Trial 2 to discard any possible undesirable effects of blueberry inclusion on digestibility. The analyzed chemical composition of the CON and BLU diets is presented in Table 1. Analyses were conducted on a representative pooled sample, composed of multiple pouches of wet food from the batch used in the trial, for each diet.

Six dogs were assigned to the CON diet and 6 to the BLU diet. Nutrient digestibility was assessed based on the recommendations of the Association of American Feed Control Officials (AAFCO) [29], following a total fecal collection protocol. After 2 weeks of adaptation to the diets, total feces corresponding to 5 days were collected using the marker-to-marker method [30]. For that, ferric oxide (Fe_2_O_3_) was added to the food on the first day of collection and again 5 days later. Total feces were collected from the time feces turned red (included), until they turned red again (not included). Feces were weighed and stored at −20 °C until analysis.

#### 2.3.2. Analytical Methods

Collected frozen feces were thawed, dried, and ground as described in [31]. All analyses were performed according to the Association of Official Agricultural Chemists (AOAC) methods [32]. Feces and diets were analyzed for moisture at 103 °C (AOAC 930.15), ash (AOAC 942.05), crude protein (AOAC 954.01), crude fat (AOAC 954.02), and neutral detergent fiber (NDF)/acid detergent fiber (ADF)/acid detergent lignin (ADL) (AOAC 973.18). Gross energy was obtained using an adiabatic calorimetric bomb (Parr 6300 Calorimeter, Parr Instrument Company, Moline, IL, USA).

#### 2.3.3. Calculations and Statistical Analysis

Dry matter was estimated as “100 − moisture (%)”, organic matter as “100 − (moisture + ash) (%)”, hemicellulose as “NDF − ADF (%)”, and cellulose as “ADF − ADL (%)”. The apparent total tract digestibility (ATTD) (%) of macronutrients was calculated as

$$\mathrm{Apparent}\;\mathrm{digestibility}=\frac{\mathrm{Ingested}-\mathrm{Excreted}}{\mathrm{Ingested}}\;\cdot \;100$$

Coefficients of digestibility were analyzed using RStudio software (version 1.4.1106), with a Student’s *t*-test analysis, after checking for normality and equal variances. Significance was set at *p* < 0.05, and trends were considered at 0.05 ≤ *p* ≤ 0.10. Data are presented as mean ± standard error of the mean (SEM).

### 2.4. Trial 3: Oxidative Balance

#### 2.4.1. Diets and Experimental Design

This trial was designed to assess the potential of the BLU diet to prevent and/or counteract oxidative stress in the animals. The trial consisted of two periods, summer (P1) and winter (P2), each lasting 56 days and divided into 2 phases. During the first phase, namely, adaptation, all dogs were fed the CON diet for 28 days. In the adaptation phase of the first period (summer), the 12 animals were introduced to a motor-driven treadmill capable of reaching 0–18 km/h and 0–20% inclination (Domyos Incline Run, model 8580105, 2019, Decathlon, Villeneuve d’Ascq, France), and 8 of the dogs were selected as runners, due to their willingness to run on it. During the adaptation phase of both periods, a standardized submaximal exercise test was designed for each runner, tailored to their physical condition as follows.

First, based on the Karvonen method (%HR_reserve_ = [(HR_work_ − HR_rest_)/(HR_max_ − HR_rest_)] × 100) [33], a target of 70% heart rate reserve (%HR_reserve_) was selected for the dogs, as it corresponds to a moderate intensity within the submaximal effort range. Then, to determine the corresponding individual heart rate of work (HR_work_), heart rate at rest (HR_rest_) and maximal heart rate (HR_max_) were assessed for each dog. Heart rates in this study were monitored using a Polar V800 Heart Rate Monitor with a Polar H7 Heart Rate Sensor attached to a Polar belt (Polar Electro Iberica, Barcelona, Spain). An area of hair was shaved and cleaned around the chest girth of the dog behind the elbows to improve belt adjustment, with a larger shaved area on the left side of the thorax at the third intercostal space, where the sensor was positioned. HR_rest_ was determined by monitoring the dogs during quiet hours of the day (14:00–18:00 h) for 30 min per dog. HR_max_ was determined as described in Section 2.4.2.

On day 28 of the period, runners performed the first submaximal exercise (S1), as described in Section 2.4.3. The 12 dogs were then assigned to 2 groups, ensuring an equal number of females, males, and runners: 6 dogs remained on the CON diet, while the other 6 changed to the BLU diet, from the following day (day 29), when the second phase started. The animals were fed their corresponding diets for 28 more days, and runners performed a second submaximal exercise (S2) on day 56 of the period, with the same %HR_reserve_ and duration as the first submaximal exercise. During this second phase, digestibility assessment was performed, but only in the first period (summer), after 2 weeks of adaptation to the diets.

As previously mentioned, trial 3 included two periods to increase the sample size. The first period was conducted from June to July, while the second period took place from February to March.

#### 2.4.2. Maximal Exercise Test

During the adaptation phase, runners performed two maximal exercise tests on alternate days to determine HR_max_, defined as the highest value recorded across the two tests. The maximal exercise test consisted in a progressive increase in exercise intensity through increases in speed and inclination, until the dogs were unable to continue the exercise willingly or their heart rate no longer increased. The procedure was adapted from the method of stages described in previous works [34]. In the present study, the dogs started running on the treadmill at approximately 4.8–5.5 km/h and 0% inclination. Then, inclination was progressively increased every 1–1.5 min, up to 20%, followed by speed increases until the exercise finished. The duration of the exercise was 13 ± 4.5 min, reaching HR_max_ at a speed of 6.4 ± 1.48 km/h and an inclination of 19.3 ± 1.55%.

#### 2.4.3. Submaximal Exercise Test

The submaximal exercise protocol was an adaptation of methods described in previous studies [27,35]. It consisted of a short warm-up during which the treadmill speed and incline were quickly increased until the animals reached the HR_work_ corresponding to 70% of their heart rate reserve. Subsequently, the dogs were to maintain this workload, which typically ranged from approximately 4.8 to 6 km/h and 4 to 18% inclination, depending on the individual fitness level, for a minimum duration of 15 min. The duration of the exercise session was determined by the dog’s willingness to run, with a maximum of 31 min for the entire exercise. Part of the daily ration was offered intermittently and in small amounts during the exercise session, solely as a motivational tool, subtracting this amount from the daily ration. Both submaximal exercise sessions at the end of phase 1 (S1) and 4 weeks after (S2) were standardized for each dog, maintaining the same target HR_work_ and duration to ensure equivalent workloads.

#### 2.4.4. Analytical Methods

On the days when submaximal exercises were performed (S1 and S2), blood samples from the runners were collected from the jugular vein into lithium heparinized tubes before (t0), immediately after (t1), and 24 h after (t2) the test. These time points were selected to capture both the acute response to exercise (t1) and the subsequent recovery phase (t2). A single blood sample (t0) from the dogs that did not run was also collected on the same day. Plasma was obtained from the samples after centrifugation at 2054× *g* for 10 min (Nahita Blue 2615/1, Auxilab S.L., Beriáin, Spain).

Plasma urea, creatine kinase (CK), C-reactive protein (CRP), GPX, and total antioxidant status (TAS) were measured using an automated analyzer (Beckman Coulter, Olympus AU400, Krefeld, Germany). Plasma CK was measured according to the standardized method described by the International Federation of Clinical Chemistry and Laboratory Medicine (IFCC) [36]. Plasma urea was determined by the glutamate dehydrogenase method. Reagents for both CK and urea determinations were OSR (Beckman Coulter, Olympus System Reagent, O’Callaghansmills, Ireland). Plasma CRP was measured by immunoturbidimetry (reagents: Acuvet Biotech, TURBOVET Canine CRP, Zaragoza, Spain), TAS by the ABTS method (2,2′-azino-bis(3-ethylbenzthiazoline-6-sulfonic acid)) (reagents: Randox Laboratories, TAS, Crumlin, UK), and GPX by the method described by Paglia and Valentine [37] (reagents: Randox Laboratories, RANSEL, Crumlin, UK). Plasma malondialdehyde (MDA) was measured using a commercial kit (Cayman Chemical, TBARS Assay Kit, Ann Arbor, MI, USA), based on the thiobarbituric acid reactive substance assay, and results from the reaction were measured using a plate reader (Thermo Fisher Scientific, Multiskan Sky, Waltham, MA, USA).

Total polyphenol (TP) content in CON and BLU diets was determined using the Folin–Ciocalteu reagent [38]. Results were expressed in mg of gallic acid equivalents (GAE) per 100 mg of fresh matter.

#### 2.4.5. Calculations and Statistical Analysis

Data were analyzed using RStudio software (version 1.4.1106), employing ANOVA with a generalized linear mixed model (function “lmer” on R). Three separate analyses were conducted to study each objective of this trial.

First, to evaluate the effect of the designed model of oxidative stress through exercise, only runners were included in the analysis, and only data from S1 were considered, as at that moment, all the dogs were fed the same control diet. The model considered time (t0, t1, and t2) and period (P1 and P2) as fixed effects, and dogs within the same period as a random effect. The effects of time, period, and time × period interaction were tested.

Second, to assess the sole effect of ingesting a diet with blueberries for 4 weeks, all the dogs were included in the analysis, but only data from t0 were considered, since those samples were collected before exercise. To evaluate the effect of the ingestion, taxes of increase/decrease in the plasma concentration of the biomarkers at t0 between samplings (S1 and S2) were calculated (i.e., log(t0_S2_/t0_S1_)). Thus, the model considered taxes as the response variable; group (CON and BLU) and period (P1 and P2) as fixed effects; and dogs as a random effect. The effects of group, period, and group × period interaction were tested.

Third and last, to determine the impact of the blueberry ingestion on biomarkers’ responses to the oxidative stress challenge, only runners were included in the analysis, and taxes between values at t1 and t0 (t_10_) (i.e., t_10_ = log(t1/t0)), and t2 and t0 (t_20_) (i.e., t_20_ = log(t2/t0)), were calculated for each biomarker. Two separate analyses examined responses immediately after exercise (t_10_) and 24 h after (t_20_). The model considered taxes as the response variable; group (CON and BLU), sampling (S1 and S2), and period (P1 and P2) as fixed effects; and dogs within the same period as a random effect. The effects of group, sampling, period, and the interactions between them were tested.

In all cases, normality of the residues was checked, and data transformation was applied when needed. Tukey’s multiple comparison analysis was used for the comparison of means between groups when significance was achieved. Data are presented as mean ± SEM. When ratios were used for the analysis (log), results were expressed at simple ratios, without the logarithm, for better interpretation. Significance was set at *p* < 0.05, and trends were considered at 0.05 ≤ *p* ≤ 0.10.

## 3. Results

### 3.1. Trial 1: Preference

The results of the two-bowl tests are shown in Table 2. The intake ratio was greater for BLU diet and lower for CLO when compared with CON (*p* < 0.001 and *p* = 0.021, respectively); no differences were detected between CON and ALG (*p* = 0.136). When compared with CLO, the BLU intake ratio was higher (*p* < 0.001). There were no differences in first approach among the comparisons (*p* > 0.05). However, first choice favored BLU diet over CON (*p* = 0.043), ALG (*p* = 0.043), and CLO (*p* = 0.001) diets, with no significant differences for the other comparisons.

### 3.2. Trial 2: Digestibility

The ATTD coefficients were not significantly different between CON and BLU diets (*p* > 0.05), except for cellulose, with lower values for the BLU diet (*p* = 0.008) (Table 3).

### 3.3. Trial 3: Oxidative Balance

The dogs remained healthy throughout the study with no reported incidents. The average food intake for the CON and BLU diets was, respectively, 1248 ± 134 g/day and 1248 ± 90 g/day in P1 (summer), and 1348 ± 107 g/day and 1295 ± 138 g/day in P2 (winter), with no refusals recorded. The registered intakes translate into an average daily intake of 11.4 g of total polyphenols for the CON group and 13.0 g for the BLU group, yielding a difference of 1.6 g/day.

The observed HR_rest_ of the dogs during quiet hours was 75 ± 10.7 bpm (76 ± 13.9 bpm for P1 and 73 ± 6.8 bpm for P2). The HR_max_ obtained from the treadmill maximal exercises was 197 ± 19.3 bpm (198 ± 16.0 bpm for P1 and 195 ± 23.3 bpm for P2). These values of HR_rest_ and HR_max_ resulted in an HR_work_ of 160 ± 13.3 bpm (162 ± 11.0 bpm for P1 and 158 ± 15.9 bpm for P2). For the submaximal exercises, in period 1, dogs worked at an average %HR_reserve_ of 64 ± 5.1%, with 9.0 ± 6.3 min above 70%HR_reserve_; in period 2, dogs worked at an average %HR_reserve_ of 72 ± 2.8%, with 22.5 ± 3.9 min above 70% HR_reserve_. An example of the heart rate evolution during the maximal and submaximal tests for one of the runners is shown in Supplementary Materials (Figure S1).

#### 3.3.1. Effect of the Submaximal Treadmill Exercise Model on Untrained Dogs

Table 4 shows the registered impact of the submaximal exercise on selected biomarkers. A time effect was observed for urea response, with an increase immediately after the exercise (t1) and a return to basal concentrations 24 h after (t2). Creatine kinase and MDA displayed a similar pattern, with a significant interaction effect between period and time. Specifically, there was an obvious response in period 1 that was not observed in period 2. In period 1, and similarly to urea, both CK and MDA increased after the exercise; however, in the case of CK, values returned to basal levels 24 h after, whereas MDA remained elevated. No time effect was found in period 2; moreover, concentrations at t1 were significantly higher for period 1 compared with period 2 for both biomarkers. No time response was observed for CRP and TAS, though period 1 had significantly higher values than period 2. Lastly, plasma GPX was significantly modified by the exercise, tending to decrease after exercise, and also showing higher values for period 1 compared with period 2.

#### 3.3.2. Effect of 4-Week Blueberry Ingestion on Pre-Exercise State

A significant interaction between period and group was observed for CK, with differences between groups only during period 1 (Table 5). While CK values increased over 4 weeks in the CON group, they decreased in the BLU group. A period effect was observed for CRP; however, no differences were found between the experimental groups. No other biomarkers exhibited significant differences.

#### 3.3.3. Effect of 4-Week Blueberry Ingestion on Response to a Submaximal Exercise Model

The main hypothesis of this approach was that the ingestion of blueberries could impact the response of selected biomarkers to an exercise model intended to induce oxidative stress. This was expected to result in a sampling per group interaction effect; however, no such effect was observed for any of the evaluated parameters when the variation between pre-exercise (t0) and post-exercise (t1) was analyzed (Table 6). For CK, a triple interaction between sampling, group, and period was observed, but a subsequent comparison of means did not show any relevant result for the hypothesis. A graphical representation of this interaction is available in Supplementary Materials (Figure S2), showing that some dogs in the CON group seemed to be more sensitive to the exercise performed at the end of phase 1 (S1) in period 1 (summer), when all the dogs were fed the control diet, due to random variation. In the case of MDA, the overall increment between t0 and t1 in period 1 was significantly higher than that in period 2 (1.20 ± 0.064 vs. 1.04 ± 0.059, respectively). For GPX, differences between samplings were significant but not biologically relevant.

When the variation between t0 and t2 was analyzed, no clear effect of blueberry ingestion was observed (Table 7). Similar to the previous analysis, CK and additionally TAS showed a triple interaction between sampling, group, and period. A graphical representation of these interactions is available in Supplementary Materials (Figure S3). In the case of CK, the response of the BLU group differed between samplings in period 1, although it continued to be equal to the response of the control group. For TAS, the triple interaction suggests differences in the response of some dogs from the BLU group at sampling 1, when all animals were fed the control diet, due to random variation. However, changes were close to 0. In the case of GPX, slight differences between samplings were again detected.

## 4. Discussion

### 4.1. Selection of Blueberries as a Candidate Ingredient with Antioxidant Potential

Diet preference in dogs can be influenced by various factors including odor, texture, and serving temperature [39]. In this study, the diet with blueberries was preferred, likely influenced by the texture (whole fruit vs. powder) and the probably higher sweetness of these fruits. The physical characteristics of the food have been shown to impact preference in dogs and cats. Dogs preferred kibbles including sugar cane fiber with a large particle size over the same food with smaller particles [40], or cross-shaped over round-shaped kibbles [41]. Unlike cats, dogs possess sweet receptors and can differentiate between sugar concentrations [42,43]. The inclusion of brown algae did not negatively impact preference. Previous studies on microalgae in dog diets have shown mixed effects, with intake ratios either decreasing [44] or increasing/not changing [45,46], suggesting that factors such as flavor (fishy smell) or presentation form (powdered or integrated into the kibble) influence acceptability. Conversely, powdered clove reduced intake compared with the control and blueberry diets, possibly due to its intense odor, similar to findings with essential oil blends [47,48]. However, diets containing other spices such as curcumin or essential oil mixes including clove did not alter body weight after weeks of feeding, suggesting no significant impact on overall diet acceptance [49,50].

The addition of blueberries did not negatively impact the apparent digestibility of macronutrients, except for a reduction in cellulose digestibility. This change is unlikely to be due to an increase in dietary cellulose from the inclusion of blueberries, as both diets had similar composition. Blueberries are a source of phenolic compounds that can form complexes with dietary fiber and digestive enzymes such as cellulases, potentially lowering its degradability [51,52]. Polyphenols can also influence the composition of the gut microbiota [53]. In ruminants, the addition of tannins, a type of polyphenol, has been shown to reduce the abundance of cellulolytic bacteria [54]. It is possible that specific bacteria with cellulolytic activity in dogs may have been similarly affected by the additional polyphenols, but further studies are needed to test this hypothesis.

### 4.2. Utility of a Submaximal Exercise Model and Selected Biomarkers in Oxidative Stress Assessment

The impact of exercise on oxidative status in canines has been predominantly studied in athletic dogs engaging in various exercise modalities [55,56,57,58]. Exercise has been linked to increased reactive oxygen metabolites (ROM) [56], lipid peroxidation [27,59], and DNA oxidation [23]. While many studies report elevated indices of oxidative damage and decreased antioxidant defenses, results may vary due to factors like exercise type and intensity, previous nutrition, or environmental conditions. The level of training might also play an important role, as regular exercise may induce a hormesis effect [60]. Exploring models involving less athletic breeds, such as research Beagles, offers valuable insights into this research field and can serve as tools for evaluating feed additives like antioxidants.

In this study, urea concentration increased immediately after exercise and returned to baseline within 24 h, similar to earlier reports [58,61,62]. As a by-product of protein metabolism, elevated urea may reflect muscle catabolism and associated reduced renal blood flow [61]. Sampling time can also influence urea levels, as no changes were observed 3 or 6 h after exercise in dogs [26,63]. Exercise type (e.g., intensity, duration, frequency) may further affect outcomes, as sled dogs showed no significant urea changes immediately after a short, high-intensity exercise [64].

Creatine kinase is an enzyme released into circulation after disruption of myocyte membranes, and it is often indicative of muscle damage [65]. Its activity increases with intense exercise, with sled dogs showing significant elevations during and shortly after endurance races [61,65,66]. Increases in circulating CK may reflect the intensity of the exercise and, indirectly, cellular oxidative damage, as it has been correlated with products of lipid peroxidation such as isoprostanes [59]. Creatine kinase increased 2-fold after exercise in period 1 (summer), returning to baseline within 24 h, suggesting transient muscle disturbance without permanent damage [67]. Similar responses were reported in other studies with dogs [26,58,68]. However, no differences in CK were found in period 2 (winter).

Malondialdehyde responded similarly to CK, with changes detected only in period 1, although increases persisted for 24 h, as observed in studies with Beagles during submaximal treadmill exercise [27]. Malondialdehyde is a product of the degradation and decomposition of polyunsaturated fatty acids by ROS, and it is highly correlated with isoprostanes [69]. However, registered values were only slightly above the normal range for healthy adult dogs (5–11 µmol/L) [70]. Other studies with trained canicross dogs from different breeds [58] and Foxhounds [26] showed no significant MDA changes after exercise.

The similar patterns observed for CK and MDA with both responding only in period 1 might have been influenced by the environmental temperature. The first period (summer, June–July) featured an average outdoor temperature of 24.1 ± 2.5 °C, with several days exceeding maximal temperatures of 29.5 °C. The second period (winter, February–March) had an average temperature of 11.3 ± 1.7 °C. The United States Department of Agriculture (USDA) Animal Welfare Act recommends that temperature should not rise above 29.5 °C for dogs housed indoor, nor fall below 10 °C if the animals are not acclimated to low temperatures [71]. High temperatures are a source of oxidative stress, with heat stress used as a model to study the protective effects of antioxidants [72,73]. Dogs performed the exercise in an indoor room that received air conditioning from an adjacent room. However, temperature differences between periods in the exercising room were still likely, with the higher temperature during summer potentially acting as an oxidant stressor in addition to the exercise. Performing exercise in hot conditions resulted in increased oxidative stress assessed by elevated lipid peroxidation markers in humans and horses [74,75,76]. Moreover, as dogs have few sweat glands, one of their strategies to maintain thermoneutrality is panting [77]. Similar to birds, panting produces greater excretion of CO_2_ compared with its production, leading to hypocapnia and respiratory alkalosis. As a compensatory mechanism, secondary metabolic acidosis can occur, which may also contribute to increase oxidative stress [78,79]. Although submaximal sessions were designed so that animals run at 70% HR_reserve_, dogs struggled to reach the established parameters during summer, with a lower average heart rate reserve of work and less time above the marked 70%. Conversely, the same dogs were able to meet the set parameters during the winter period. This worse execution also points to the effect of temperature, as other studies have shown that dogs running on a treadmill had better performance when cooling aids were used [80]. Interestingly, the response of CK and MDA only during the summer period might suggest that the designed exercise per se was not enough to induce a change in these biomarkers, but the presence of an additional stressor such as heat provoked a response. Changes in winter were still expected, as moderate exercise (70% HR_reserve_) is similar to previous research with Beagles (75% HR_reserve_), where markers of oxidation such as MDA varied [27].

Lastly, the activity levels of the dogs around the kennel and outdoor area may have been influenced by the environmental temperature, impacting their response to the submaximal exercise. Temperature can influence activity and behavior, with dogs being less active during the warmest hours of the day in summer to maintain thermal neutrality [81]. Owners also perceived decreased exercise intensity and duration in hot conditions [82]. Dogs might have been less active in period 1 to avoid heat stress, while cooler temperatures in period 2 likely encouraged greater activity. Periods of restricted activity have been shown to reduce endurance in dogs [83], whereas activity improves aerobic capacity [68]. The hypothesized lower activity levels in period 1 may have contributed to the greater impact of the submaximal session during the summer period.

As the most sensitive acute phase protein (APP) in dogs, CRP can be a marker of inflammation triggered by injuries, infections, stress, or neoplasia [84]. Exercise can release APP due to muscle, joint, or skeletal injuries (including microinjuries) and/or glycogen depletion in exercising muscles [85]. The intensity of the exercise relative to the dog’s fitness level may determine CRP response, as sled dogs showed significant increases in CRP when running long distances [62,66,86], but not so much in shorter runs [87]. As CRP usually changes dramatically 4–6 h after an inflammatory trigger [84], sampling immediately after exercise may have precluded observing significant changes. The marked period effect observed in CRP, with values more than 6-fold higher in period 1 (summer), could be due to environmental differences between summer and winter, as heat stress has been linked to APP activation to protect tissues from injury [88]. It is also relevant to highlight that, despite the observed differences, values were always within the general reference interval (<20 mg/L).

Glutathione peroxidase, an antioxidant enzyme that primarily catalyzes the reduction of H_2_O_2_ and organic hydroperoxides, tended to decrease immediately and 24 h after exercise, consistent with enzyme consumption due to the expected increased ROS. While maximal efforts can induce GPX reductions [89], the type and intensity of the exercise, as well as previous training, might vary the response. The concentration of GPX was reported to decrease in sled dogs participating in a long-distance race [55], but not in a shorter one [59]. Similarly, trained canicross dogs and humans cycling at moderate intensity showed no significant GPX changes [58,90]. A period effect was identified, with significantly higher average GPX values during period 1 (summer), which may be attributable to the higher temperature during summer [91].

Total antioxidant capacity measured as TAS, also known as Trolox equivalent antioxidant capacity (TEAC), measures the overall antioxidant power of a sample, accounting for synergistic interactions among various antioxidants [92]. Exercise can lower antioxidant capacity in athletic dogs [24,56]. However, other studies have shown stable levels after exercise [57,59], aligning with our findings. The observed decrease in GPX, and potential reductions of other endogenous antioxidants after exercise, may have been offset by hypothetical increases in components with antioxidant activity such as uric acid, whose rise after exercise has been reported in dogs [25,57,93]. A significant period effect in this study revealed higher TAS concentration during period 1, with elevated GPX levels likely contributing to this result. The complex relations behind TAS behavior emphasize the need for multiple biomarker analyses rather than relying on a single parameter.

Possible improvements to the model that would deserve future consideration include a larger sample size and tighter control of environmental variables to better account for their potential influence on animal responses. The inclusion of additional biomarkers, such as lactate, could also be beneficial for more accurately characterizing exercise intensity. Furthermore, increasing the target %HR_reserve_ to 75–80% could also be desirable to intensify the physiological challenge, potentially eliciting more pronounced biomarker responses and enhancing the model’s sensitivity to detect modulatory effects when dietary interventions are applied.

### 4.3. Effect of the 4-Week Ingestion of Blueberries on Resting State (Before the Exercise)

Blueberries are natural ingredients rich in antioxidant polyphenols, particularly anthocyanins, which have been associated with various health benefits, including improvements in inflammation, oxidation, and metabolic syndrome markers [94]. Considering the total polyphenol content of both experimental diets and the average food intake, we estimate that animals receiving the BLU diet consumed approximately 1.6 g more total phenolics per day than those on the CON diet. Human studies have demonstrated increased serum antioxidant status following the ingestion of a single intake of 100 g of freeze-dried blueberry powder (equivalent to 2.8 g of total phenolics), compared with a control supplement without polyphenols [95]. Similarly, the supplementation of 45 g of a freeze-dried blueberry powder daily in humans for 6 weeks (equivalent to 1.6 g/day of total phenolics) resulted in decreased ROS in the blood and monocytes [96]. In the present study, however, 4 weeks of blueberry ingestion did not significantly influence most of the analyzed biomarkers. Other studies supplementing Alaskan huskies with 20 g of blueberries daily (compared with approximately 38 g/day in our study) for 2 months also failed to evidence changes in total antioxidant capacity before exercise [25]. Conversely, feeding dogs kibbles supplemented with a blueberry and grape extract for 75 days resulted in the upregulation of antioxidant enzyme gene expression [97]. Although the precise intake of total phenolics was not stated in that study, it can be estimated to be around 60–80 g per day. These values greatly exceed the inclusion level used in the present study and may explain the lack of response observed in the selected biomarkers.

Despite the lack of significant changes in most of the biomarkers analyzed, an interaction between period and group was observed for CK, with decreased concentration after 4 weeks of blueberry administration, but only during period 1 (summer). The high temperatures in this period, as previously discussed, may have introduced an additional source of oxidative stress, potentially allowing blueberries to exhibit their protective effect against oxidative stress and muscle damage. These findings suggest that including 3% blueberries in the tested wet diet for 4 weeks might help prevent elevated CK levels, particularly under heat stress conditions. Healthy dogs fed a canned diet supplemented with hempseed cake, a rich source of polyphenols, for 30 days showed lower blood CK concentrations, compared with supplementation with swine tallow [98]. Although authors suggested that this difference might be due to the content of polyunsaturated fatty acids, the high content of polyphenols might have also contributed to the decreased CK. However, other authors have not reported significant changes in CK levels in dogs supplemented with blueberries or bilberries, compared with non-supplemented controls [25].

### 4.4. Effect of the 4-Week Ingestion of Blueberries on the Response Against a Submaximal Exercise Model

Research on the efficacy of blueberries in attenuating exercise-induced oxidative stress in humans has shown that its supplementation reduced levels of markers of lipid peroxidation [99,100], improved recovery rates [101], and increased total antioxidant status [102] after exercise. To the authors’ knowledge, only one study has examined blueberry supplementation in exercising dogs, specifically sled Alaskan huskies [25]. This study did not demonstrate a protective effect on muscle damage but showed an increase in the level of antioxidants available after exercise in the group fed 20 g of blueberries daily for 2 months, compared with non-supplemented dogs that did not exercise. However, no differences were found when compared with non-supplemented dogs that underwent exercise.

In the present study, the inclusion of blueberries in the diet (3%) for 4 weeks did not significantly influence biomarker responses following a submaximal treadmill exercise. Blueberry supplementation did not mitigate the increase in CK after exercise, consistent with previous findings [25]. While the anti-inflammatory and antioxidant properties of blueberries have been described, factors such as inclusion level, exercise type, and individual variability can influence the outcome. For instance, some authors only observed the effects of the supplementation after exercise, when the production of reactive oxygen species is supposed to increase [25]. However, insufficient exercise intensity can prevent the manifestation of potential benefits. In this study, the treadmill exercise may not have been intense enough to cause a great impact on biomarkers, even though the target HR of work based on an %HR_reserve_ of 70%, particularly during winter period, was similar to previous research where effects on lipid peroxidation were observed [27]. Additionally, although the dogs were untrained, they could roam freely and play, so a certain degree of activity might have affected the results, unlike other studies where dogs were housed in cages [27]. However, changes induced by the submaximal exercise were found in period 1 in several biomarkers, but no effect of the inclusion of blueberries in the diet was detected. The selection of biomarkers can also be decisive when identifying the effects of the supplementation, as daily intake of 150 g of blueberries in humans did not suppress the exercise-induced increase in F2-isoprostanes but did reduce lipid hydroperoxide elevation [99].

Another potential explanation for the lack of effect is an insufficient period of administration. Some studies in dogs investigating supplementation effects on exercise often span longer periods, ranging from 2 months to 80 days [25,26,27]. However, extended supplementation does not always guarantee effects after exercise [26]. In contrast, similar to the length of the present study, 1 month of supplementation was enough to see an impact of a mix of antioxidants in sled dogs after a race [23]. Lastly, in humans, 7 days of blueberry consumption attenuated the amount of lipid hydroperoxides after exercise, without affecting isoprostane levels [99].

Despite evidence supporting the potential of blueberries and anthocyanins to protect against exercise-induced oxidative stress, this study did not demonstrate a protective effect from the inclusion of 3% whole blueberries in the diet of dogs over a 4-week period. Possible explanations include the source and inclusion level of blueberries, small sample size, or limitations in the sensitivity of the experimental methods, which may have been insufficient to detect its antioxidant potential. Further research is needed to investigate these factors and elucidate the underlying mechanisms of action.

## 5. Conclusions

The inclusion of blueberries, a natural source of flavonoids, in a wet diet for dogs improved its preference without negatively impacting apparent digestibility. Feeding the blueberry-enriched diet for 4 weeks did not significantly alter biomarkers of muscle damage, inflammation, or antioxidant status following submaximal treadmill exercise. However, the observed reduction in creatine kinase levels in the group fed blueberries during the 4-week summer period suggests a potential protective effect against heat stress, requiring further investigation. Additionally, the submaximal treadmill exercise model used in this study influenced various biomarkers, particularly under high-temperature conditions, highlighting their potential as a valuable tool for evaluating the antioxidant bioactivity of feed additives or functional ingredients.

## Figures and Tables

**Table 1:** Analyzed chemical composition of CON and BLU diets (as fed).

	CON	BLU
Dry matter (%)	16.4	16.8
Crude protein (%)	9.14	9.58
Crude fat (%)	3.32	3.45
Ash (%)	1.65	1.65
Hemicellulose (%)	0.54	0.69
Cellulose (%)	0.17	0.16
Gross energy (kcal/kg)	915.1	954.3
Total polyphenols (mg GAE/100 mg)	0.88	1.02

Notes: CON = control wet diet; BLU = control diet including whole blueberries (3%); and GAE = gallic acid equivalents.

**Table 2:** Intake ratio (%), first approach as the number of tests where diet A/B was approached first, and first choice as the number of tests where diet A/B was the first the dogs consumed food from for more than 15 s consecutively (*n* = 12).

Comparison		Intake Ratio (%)			First Approach			First Choice		
Diet A	Diet B	Diet A	Diet B	*p*-Value	Diet A	Diet B	*p*-Value	Diet A	Diet B	*p*-Value
CON	ALG	60.3	39.7	0.136	11	13	0.773	15	9	0.149
CON	CLO	66.1	33.9	0.021	11	13	0.773	15	9	0.149
CON	BLU	31.2	68.8	<0.001	11	13	0.773	8	16	0.043
ALG	CLO	60.1	39.9	0.185	11	13	0.773	13	11	0.773
ALG	BLU	35.8	64.2	0.099	14	10	0.387	8	16	0.043
CLO	BLU	21.2	78.8	<0.001	15	9	0.149	6	18	0.001

Notes: CON = control diet; BLU = control diet including whole blueberries (3%); CLO = control diet including powdered clove (0.45%); and ALG = control diet including powdered *Fucus* algae (1.5%). All diets were wet diets.

**Table 3:** Coefficients of apparent total tract digestibility (%) for CON and BLU diets (*n* = 6). Data presented as mean ± SEM.

	CON	BLU	*p*-Value
Dry matter (%)	85.7 ± 0.67	85.1 ± 0.30	0.449
Organic matter (%)	88.8 ± 0.64	88.2 ± 0.34	0.435
Crude protein (%)	91.6 ± 0.54	91.4 ± 0.31	0.686
Crude fat (%)	92.1 ± 0.19	92.0 ± 0.50	0.803
Hemicellulose (%)	76.0 ± 3.31	84.1 ± 1.59	0.053
Cellulose (%)	49.8 ± 5.52	24.0 ± 5.55	0.008
Energy (%)	89.1 ± 10.54	88.8 ± 0.21	0.687

Notes: CON = control wet diet; BLU = control diet including whole blueberries (3%).

**Table 4:** Impact of the submaximal exercise on the response of selected biomarkers. Only data from first submaximal exercise (sampling 1) are included, as all the dogs were fed the same control diet (*n* = 16 *). Data are expressed as means ± SEM.

	Period (P)	Time-Point (t)				*p*-Values		
		t0	t1	t2		P	t	P × t
Urea	P1	37.3 ± 1.70	41.0 ± 2.02	35.8 ± 2.28	38.0 ± 1.20	0.346	<0.001	0.634
	P2	36.9 ± 1.81	38.8 ± 1.96	33.8 ± 1.59	36.5 ± 1.08			
		37.1 ± 1.20 ^b^	39.9 ± 1.39 ^a^	34.8 ± 1.36 ^b^				
CK	P1	168 ± 29.9 ^b^	335 ± 60.1 ^aA^	167 ± 17.5 ^b^	215 ± 26.0	<0.001	0.001	0.009
	P2	146 ± 7.5	146 ± 9.5 ^B^	121 ± 6.3	138 ± 5.0			
		156 ± 14.2	227 ± 35.9	144 ± 10.8				
CRP	P1	8.36 ± 3.570	8.11 ± 3.492	6.60 ± 1.979	7.69 ± 1.719	0.004	0.261	0.930
	P2	1.24 ± 0.278	1.12 ± 0.288	1.31 ± 0.259	1.22 ± 0.153			
		4.80 ± 1.959	4.62 ± 1.918	3.95 ± 1.182				
MDA	P1	8.28 ± 0.729 ^b^	11.61 ± 0.923 ^aA^	10.94 ± 0.931 ^a^	10.36 ± 0.569	0.166	0.026	0.004
	P2	10.14 ± 1.183	9.83 ± 0.924 ^B^	9.92 ± 0.750	9.96 ± 0.535			
		9.27 ± 0.735	10.72 ± 0.671	10.43 ± 0.592				
GPX	P1	34,365 ± 3995.9	31,569 ± 2961.6	31,210 ± 3405.4	32,417 ± 1967.6	0.012	0.045	0.202
	P2	25,318 ± 1407.0	24,545 ± 1611.9	24,929 ± 1286.3	24,930 ± 797.9			
		29,841 ± 2356.3 ^x^	27,823 ± 1823.2 ^y^	28,069 ± 1936.4 ^y^				
TAS	P1	1.12 ± 0.035	1.14 ± 0.031	1.13 ± 0.015	1.13 ± 0.016	<0.001	0.348	0.747
	P2	0.98 ± 0.024	1.03 ± 0.011	1.01 ± 0.015	1.00 ± 0.011			
		1.05 ± 0.027	1.08 ± 0.021	1.07 ± 0.019				

Notes: P = experimental period; P1 = period 1; P2 = period 2; t = time; t0 = just before submaximal exercise; t1 = just after submaximal exercise; and t2 = 24 h after submaximal exercise. Units: creatine-kinase/CK (U/L), urea (mg/dL), C-reactive protein/CRP (mg/L), malondialdehyde/MDA (µM), glutathione peroxidase/GPX (U/L), total antioxidant status/TAS (mmol/L). Superscripts ab in a row indicate statistical differences between times within a period (*p* < 0.05). Superscripts AB in a column indicate statistical differences between periods within a time (*p* < 0.05). Superscripts xy in a row indicate statistical trends between times within a period (*p* < 0.10). Cells with gray background indicate average values per period (gray column) or per time point (gray rows). * Abnormal values excluded from analysis associated with laboratory analysis: CK—one from P1-t0, two from P1-t1; MDA—one from P1-t0; GPX—one from P1-t1.

**Table 5:** Effect of a 4-week ingestion of a diet with 3% blueberries on selected biomarkers. Values represent variation between samplings, 4 weeks apart (t0 value in sampling 2/t0 value in sampling 1) (*n* = 12 *). Data are expressed as means ± SEM.

	Group (g)	Period (P)			*p*-Values		
		P1	P2		P	g	P × g
Urea	CON	1.08 ± 0.075	0.96 ± 0.063	1.02 ± 0.050	0.051	0.700	0.633
	BLU	1.17 ± 0.138	0.94 ± 0.019	1.05 ± 0.075			
		1.12 ± 0.076	0.95 ± 0.031				
CK	CON	1.28 ± 0.260 ^A^	1.08 ± 0.144	1.19 ± 0.153	0.110	0.059	0.021
	BLU	0.61 ± 0.045 ^bB^	1.16 ± 0.153 ^a^	0.89 ± 0.128			
		1.01 ± 0.187	1.12 ± 0.100				
CRP	CON	0.85 ± 0.177	1.43 ± 0.126	1.14 ± 0.135	0.011	0.990	0.991
	BLU	0.87 ± 0.191	1.45 ± 0.175	1.16 ± 0.152			
		0.86 ± 0.124	1.44 ± 0.103				
MDA	CON	1.00 ± 0.101	0.83 ± 0.075	0.92 ± 0.066	0.465	0.598	0.448
	BLU	0.99 ± 0.134	1.00 ± 0.137	1.00 ± 0.092			
		1.00 ± 0.078	0.91 ± 0.079				
GPX	CON	1.01 ± 0.062	1.04 ± 0.017	1.02 ± 0.031	0.366	0.925	0.752
	BLU	0.99 ± 0.061	1.05 ± 0.051	1.02 ± 0.039			
		1.00 ± 0.042	1.04 ± 0.026				
TAS	CON	0.92 ± 0.023	1.02 ± 0.035	0.97 ± 0.024	0.156	0.207	0.216
	BLU	1.02 ± 0.057	0.99 ± 0.017	1.00 ± 0.028			
		0.97 ± 0.032	1.00 ± 0.019				

Notes: P = experimental period; P1 = period 1; P2 = period 2; g = group; BLU: control diet including whole blueberries (3%); CON: control wet diet; CK: creatine kinase; CRP: C-reactive protein; GPX: glutathione peroxidase; MDA: malondialdehyde; and TAS: total antioxidant status. Superscripts ab in a row indicate statistical differences between periods within a group (*p* < 0.05). Superscripts AB in a column indicate statistical differences between groups within a period (*p* < 0.05). Cells with gray background indicate average values per group (gray column) or per period (gray rows). * Abnormal values excluded from analysis associated with laboratory analysis: CK—two from BLU-P1, one from CON-P2, two from BLU-P2; MDA—one from BLU-P1.

**Table 6:** Impact of a 4-week ingestion of a diet with 3% blueberries on the immediate response of selected biomarkers in front of a submaximal exercise. Values represent variation between the blood samples taken just after (t1) and before (t0) the exercise. The table shows changes induced by two submaximal exercises 4 weeks a part (S1 on day 28 and S2 on day 56) (*n* = 8 *). Data are expressed as means ± SEM.

	Group (g)	Sampling (S)			*p*-Values				
		S1	S2		S	g	P	S × g	S × g × P
Urea	CON	1.09 ± 0.020	1.05 ± 0.062	1.07 ± 0.032	0.262	0.536	0.545	0.931	0.873
	BLU	1.06 ± 0.019	1.01 ± 0.059	1.04 ± 0.030					
		1.08 ± 0.014	1.03 ± 0.042						
CK	CON	1.59 ± 0.412	1.48 ± 0.242	1.52 ± 0.215	0.059	0.035	0.011	0.181	0.006
	BLU	1.14 ± 0.151	1.21 ± 0.258	1.17 ± 0.132					
		1.35 ± 0.207	1.38 ± 0.176						
CRP	CON	0.88 ± 0.075	1.05 ± 0.099	0.97 ± 0.064	0.641	0.971	0.856	0.300	0.856
	BLU	0.97 ± 0.055	0.96 ± 0.136	0.97 ± 0.071					
		0.93 ± 0.046	1.01 ± 0.082						
MDA	CON	1.15 ± 0.099	1.18 ± 0.087	1.17 ± 0.064	0.228	0.290	0.036	0.126	0.215
	BLU	1.17 ± 0.099	0.97 ± 0.072	1.06 ± 0.063					
		1.16 ± 0.068	1.07 ± 0.061						
GPX	CON	0.99 ± 0.044	1.04 ± 0.021	1.01 ± 0.024	0.033	0.064	0.545	0.492	0.119
	BLU	0.92 ± 0.036	1.00 ± 0.019	0.96 ± 0.022					
		0.96 ± 0.029	1.02 ± 0.015						
TAS	CON	1.05 ± 0.032	1.03 ± 0.014	1.04 ± 0.017	0.801	0.967	0.581	0.449	0.358
	BLU	1.03 ± 0.037	1.05 ± 0.024	1.04 ± 0.021					
		1.04 ± 0.024	1.04 ± 0.014						

Notes: S = sampling; S1 = sampling 1; S2 = sampling 2 (4 weeks apart from sampling 1); g = group; P = experimental period; BLU: control diet including whole blueberries (3%); CON: control wet diet; CK: creatine kinase; CRP: C-reactive protein; GPX: glutathione peroxidase; MDA: malondialdehyde; and TAS: total antioxidant status. Cells with gray background indicate average values per group (gray column) or per sampling (gray rows). * Abnormal values excluded from analysis associated with laboratory analysis: CK—two from CON-S1, one from BLU-S1, three from BLU-S2; MDA—one from BLU-S1; GPX—one from BLU-S1.

**Table 7:** Impact of a 4-week ingestion of a diet with 3% blueberries on the late response of selected biomarkers in front of a submaximal exercise. Values represent variation between the blood samples taken 24 h after (t2) and just before the exercise (t0). The table shows changes induced by two submaximal exercises 4 weeks a part (S1 on day 28 and S2 on day 56) (*n* = 8 *). Data are expressed as means ± SEM.

	Group (g)	Sampling (S)			*p*-Values				
		S1	S2		S	g	P	S × g	S × g × P
Urea	CON	0.90 ± 0.047	0.82 ± 0.068	0.86 ± 0.041	0.115	0.156	0.501	0.842	0.599
	BLU	0.98 ± 0.034	0.90 ± 0.045	0.94 ± 0.029					
		0.94 ± 0.029	0.86 ± 0.041						
CK	CON	1.21 ± 0.231	1.25 ± 0.292	1.23 ± 0.177	0.051	0.554	0.390	0.047	0.015
	BLU	0.78 ± 0.083	1.38 ± 0.177	1.03 ± 0.122					
		1.01 ± 0.137	1.31 ± 0.180						
CRP	CON	1.11 ± 0.140	1.01 ± 0.065	1.06 ± 0.079	0.896	0.629	0.194	0.530	0.853
	BLU	1.13 ± 0.179	1.16 ± 0.174	1.14 ± 0.121					
		1.12 ± 0.110	1.08 ± 0.091						
MDA	CON	1.09 ± 0.098	1.11 ± 0.132	1.10 ± 0.079	0.205	0.884	0.656	0.201	0.102
	BLU	1.18 ± 0.082	0.96 ± 0.062	1.06 ± 0.057					
		1.13 ± 0.063	1.04 ± 0.073						
GPX	CON	0.98 ± 0.027	1.08 ± 0.028	1.03 ± 0.023	0.001	0.188	0.841	0.477	0.768
	BLU	0.92 ± 0.033	1.06 ± 0.043	0.99 ± 0.032					
		0.95 ± 0.022	1.07 ± 0.025						
TAS	CON	1.00 ± 0.033	0.98 ± 0.024	0.99 ± 0.020	0.201	0.097	0.261	0.667	<0.001
	BLU	1.05 ± 0.043	1.01 ± 0.026	1.03 ± 0.025					
		1.02 ± 0.027	1.00 ± 0.017						

Notes: S = sampling; S1 = sampling 1; S2 = sampling 2 (4 weeks apart from sampling 1); g = group; P = experimental period; BLU: control diet including whole blueberries (3%); CON: control wet diet; CK: creatine kinase; CRP: C-reactive protein; GPX: glutathione peroxidase; MDA: malondialdehyde; TAS: total antioxidant status. Cells with gray background indicate average values per group (gray column) or per sampling (gray rows). * Abnormal values excluded from analysis associated with laboratory analysis: CK—one from BLU-S1, one from CON-S2, three from BLU-S2; CRP—one from CON-S2, one from BLU-S2; MDA—one from BLU-S1.

## Data Availability

The data presented in this study are available on request from the corresponding author. The data are not publicly available due to privacy restrictions.

## Supplementary Materials

The following supporting information can be downloaded at https://www.mdpi.com/article/10.3390/ani15101502/s1: Figure S1: Example of the heart rate evolution with time for one of the dogs during maximal (A) and submaximal (B) treadmill sessions; Figure S2: Graphical representation of the triple interaction effect (*p* = 0.006) for creatine kinase (CK) response against submaximal exercise; Figure S3: Graphical representation of the triple interaction effect for responses of creatine kinase (CK) (A, *p* = 0.015) and total antioxidant status (TAS) (B, *p* < 0.001) against submaximal exercise.

## Abbreviations

The following abbreviations are used in this manuscript:

ADF	acid detergent fiber
ADL	acid detergent lignin
ATTD	apparent total tract digestibility
AOAC	Association of Official Agricultural Chemists
CK	creatine kinase
CRP	C-reactive protein
GPX	glutathione peroxidase
HR	heart rate
IR	intake ratio
MDA	malondialdehyde
MER	maintenance energy requirements
NDF	neutral detergent fiber
ROS	reactive oxygen species
TAS	total antioxidant status
TP	total polyphenol
UAB	Universitat Autònoma de Barcelona
