# Supplementary material for: Effects of Blueberry Consumption on Preference, Digestibility, and Oxidative Balance in Dogs
Source: Animals (Basel). 2025 May 21;15(10):1502. doi: 10.3390/ani15101502 (PMC12108248; doi:10.3390/ani15101502)
Supplement: Supplementary file 1 [file animals-15-01502-s001.zip › animals-3621181-supplementary.pdf]

SUPPLEMENTARY MATERIALS

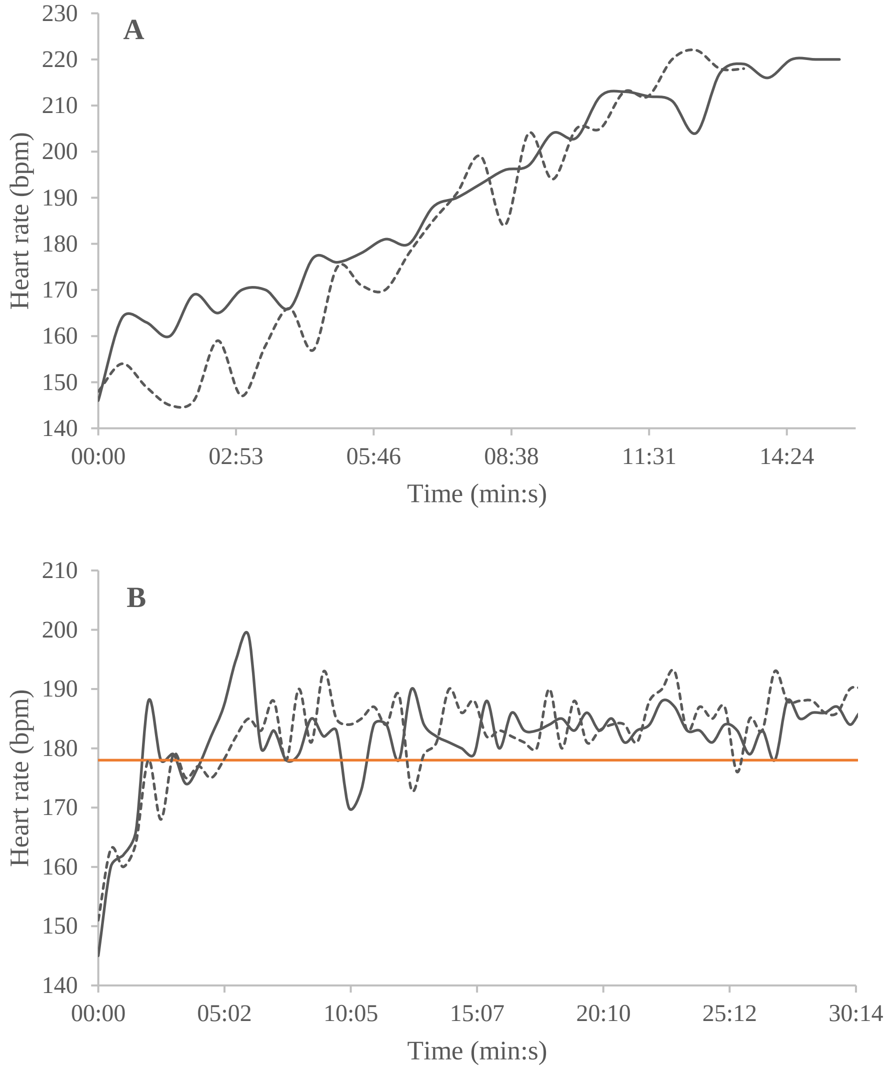

Figure S1. Example of the heart rate evolution with time for one of the dogs during maximal (**A**) and submaximal (**B**) treadmill sessions. The X axis represents the time (min:s); the Y axis represents the heart rate (bpm). **A**: the continuous line and the discontinuous line correspond to test 1 and test 2, respectively. These are both maximal exercises repeated in alternate days to determine the maximum heart rate (HR_{max}). **B**: the continuous line and the discontinuous line correspond to submaximal exercises on day 28 (sampling 1) and day 56 (sampling 2), respectively. The orange continuous line indicates the heart rate of work (HR_{work}) corresponding to 70% of the heart rate reserve ($\%HR_{reserve}$) for this example dog.

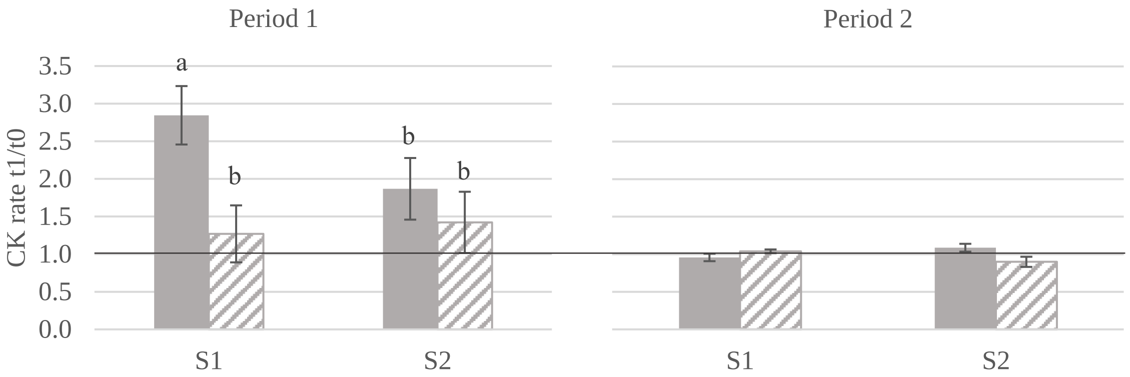

Figure S2. Graphical representation of the triple interaction effect ($p = 0.006$) for creatine kinase (CK) response against submaximal exercise. Bars represent variation between the plasma CK values pre-exercise (t0) and immediately post-exercise (t1), as mean ± SEM. Grey full bars correspond to control group, whereas patterned bars represent blueberry-fed group. Samplings 1 (S1) and 2 (S2) correspond to the submaximal exercises, performed 4 weeks apart (S1 on day 28 and S2 on day 56 of the period). Periods 1 and 2 represent summer and winter periods, respectively. Different letters (a,b) indicate statistical differences ($p < 0.05$).

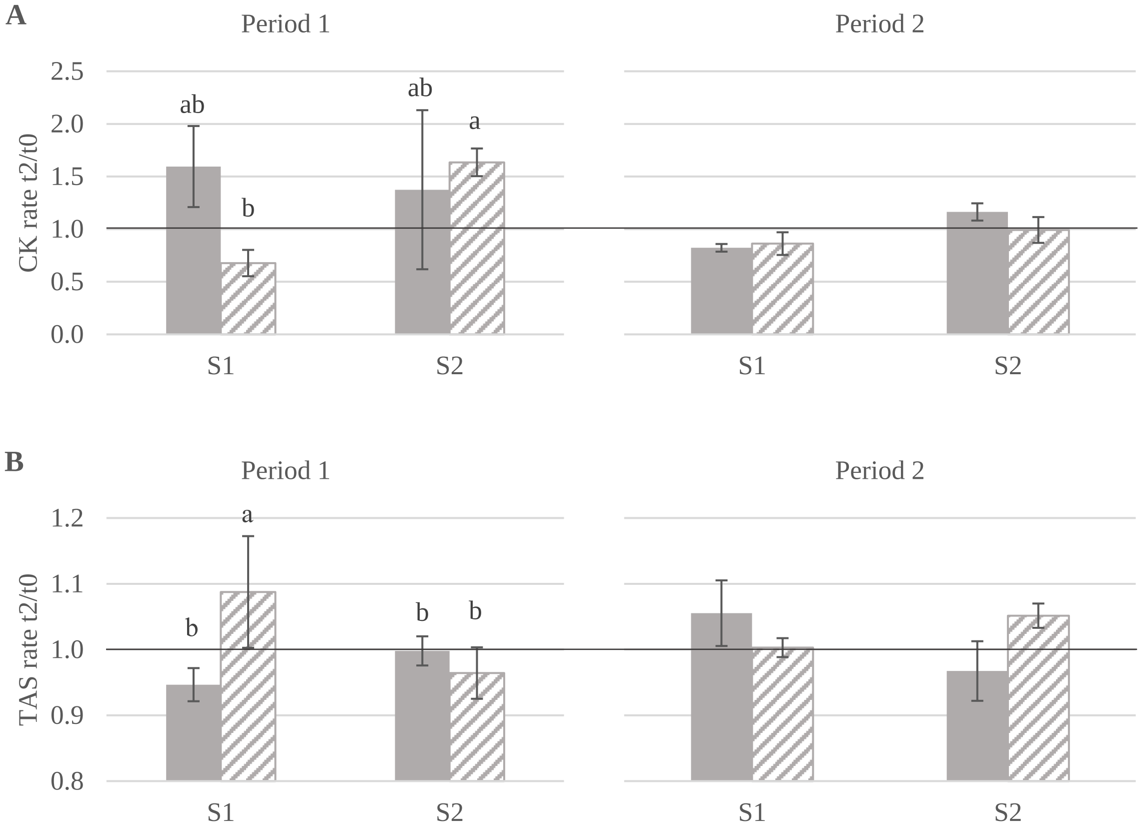

Figure S3. Graphical representation of the triple interaction effect for responses of creatine kinase (CK) (**A**, $p = 0.015$) and total antioxidant status (TAS) (**B**, $p < 0.001$) against submaximal exercise. Bars represent variation between the plasma values pre-exercise (t0) and 24 h post-exercise (t2), as mean ± SEM. Grey full bars correspond to control group, whereas patterned bars represent blueberry-fed group. Samplings 1 (S1) and 2 (S2) correspond to the submaximal exercises, performed 4 weeks apart (S1 on day 28 and S2 on day 56 of the period). Periods 1 and 2 represent summer and winter periods, respectively. Different letters (a,b) indicate statistical differences ($p < 0.05$).
